# Tobacco Usage in Uttarakhand: A Dangerous Combination of High Prevalence, Widespread Ignorance, and Resistance to Quitting

**DOI:** 10.1155/2015/132120

**Published:** 2015-07-27

**Authors:** Nathan John Grills, Rajesh Singh, Rajkumari Singh, Bradley C. Martin

**Affiliations:** ^1^The Nossal Institute for Global Health, University of Melbourne, Vic 3010, Australia; ^2^GCDWS, Chamba Hospital, Uttarakhand, India

## Abstract

*Background*. Nearly one-third of adults in India use tobacco, resulting in 1.2 million deaths. However, little is known about knowledge, attitudes, and practices (KAP) related to smoking in the impoverished state of Uttarakhand. *Methods*. A cross-sectional epidemiological prevalence survey was undertaken. Multistage cluster sampling selected 20 villages and 50 households to survey from which 1853 people were interviewed. Tobacco prevalence and KAP were analyzed by income level, occupation, age, and sex. 95% confidence intervals were calculated using standard formulas and incorporating assumptions in relation to the clustering effect. *Results*. The overall prevalence of tobacco usage, defined using WHO criteria, was 38.9%. 93% of smokers and 86% of tobacco chewers were male. Prevalence of tobacco use, controlling for other factors, was associated with lower education, older age, and male sex. 97.6% of users and 98.1% of nonusers wanted less tobacco. Except for lung cancer (89% awareness), awareness of diseases caused by tobacco usage was low (cardiac: 67%; infertility: 32.5%; stroke: 40.5%). *Conclusion*. A dangerous combination of high tobacco usage prevalence, ignorance about its dangers, and few quit attempts being made suggests the need to develop effective and evidence based interventions to prevent a health and development disaster in Uttarakhand.

## 1. Introduction

Globally, tobacco use is the second-leading cause of preventable death [1], being responsible for more than 5 million deaths annually [2]. The present burden of tobacco deaths is equally shared between developed and developing countries [1]. However, whilst tobacco consumption is declining in high-income countries, consumption is increasing in low and middle income countries (LMIC). 84% of the world's smokers now reside in LMIC countries [3] and, by 2030, 70% of tobacco-related deaths are predicted to occur in LMIC [4, 5].

In India, The World Health Organization predicts that by 2020 tobacco deaths in India may exceed 1.5 million annually [6]. More than one-third (35%) of Indian adults use tobacco [7]; however there are great variations in prevalence between the sexes, between urban and rural communities, and between different states and among different socioeconomic and cultural groups [8]. Smokeless tobacco products are the most commonly used form (21%); however over one-quarter of tobacco consumers only use smoked forms (9%), whilst one-seventh (5%) use both [7]. Smoking prevalence is much higher in men (23%) with only 3% of women smoking tobacco. Additionally, the diversity of forms of tobacco usage in India creates additional complexity for tobacco control initiatives.

The health burden of tobacco is particularly relevant for a country which is the second largest consumer of tobacco products in the world [5, 9]. The negative impacts of tobacco on health have been known by the research community for decades [10]. All forms of tobacco cause fatal and disabling health problems throughout life. However, whilst community awareness around the major tobacco related diseases has generally improved, awareness about the litany of other diseases caused by tobacco tends to be low.

Evidence shows that Tobacco use is influenced by a variety of factors, including individual attitudes and beliefs, social norms and acceptability, availability, and advertising campaigns [11]. There are also many misperceptions with regard to tobacco use, for example, that it aids concentration, suppresses appetite, reduces anxiety and tension, causes skeletal muscle relaxation, and induces feelings of pleasure [11, 12].

In Uttarakhand the Global Adult Tobacco Survey study indicates that the prevalence of tobacco use in Uttarakhand state is approximately 31%, which is the highest of all the Northern states. 44% of males use tobacco, while only 6% of females do, replicating the pattern of male-dominated tobacco use across India [7]. Another prevalence study done by Grills et al. in Tehri Garhwal District of Uttarakhand revealed that the prevalence of adult tobacco usage was similar to the national data [13]. Not only were high rates of tobacco consumption observed in Tehri Garhwal and Dehradun but also myths and misconceptions surrounding the use of tobacco were observed which reinforce consumption behaviour in communities of these two districts. Grills et al., who work in hospitals in Uttarakhand, see high numbers of tobacco related illnesses including respiratory diseases, otitis media in children, strokes, and cardiac disease.

Although preliminary surveys [13] and the GATS indicate high prevalence of tobacco usage in Uttarakhand there is limited sub-group analysis or data on the various factors influencing usage (individual attitudes and beliefs, social norms and acceptability, availability, and advertising campaigns). Given indications that Uttarakhand has a high prevalence of tobacco use, it is important to develop a more detailed understanding of tobacco usage in this rural and mountainous area of North India. This will inform the development of high quality, integrated, and cost effective tobacco control programs to decrease harm from tobacco usage in India. In particular we will undertake a detailed analysis amongst different subgroups of prevalence, knowledge of the dangers, and the importance of different factors affecting initiation and quitting behaviour.

## 2. Subjects and Methods

### 2.1. Study Design

A cross-sectional cluster randomized epidemiological mapping survey was done in two rural and mountainous districts of Uttarakhand state over a four-week period.

A power calculation suggested that a sample size of >1800 people would be adequate to estimate the outcomes in Uttarakhand (based on an ICC of 0.01). This was derived using “sampsi” and “samclus” in STATA.

From thirteen districts in Uttarakhand two were selected nonrandomly in that they represented a one-quarter of the population of Uttarakhand and also where the intervention will be taking place. In the first stage, cluster randomisation resulted in the selection of twelve out of  764 clusters (villages) from Tehri District and eighteen clusters (villages) from 1901 clusters in Dehradun district (the second district had roughly twice the population). These clusters were chosen randomly using a standard formula for probability proportionate to the size (PPS). In the second stage of the sampling, fifty households in each village were selected using standard sampling methods. Where the entire village was between 45–55 households, the entire village was interviewed. Where the cluster did not have enough households, the nearest villages were also incorporated into the cluster. Where villages had more than 55 households, we selected households by walking in a randomly determined direction and seeking to interview every second household. If households were unoccupied, and where enquiries could not determine their whereabouts, then the next household was surveyed. The response rate was extremely high with less than 15 people refusing to be interviewed, giving a response rate >99%.

### 2.2. Assessment and Outcomes

The survey tool was developed utilizing validated questions drawn from various other surveys that assessed KAP [7, 11, 14]. The two-page survey tool can be found in the Appendix. The tool was intended to measure the prevalence of tobacco usage (smoked and smokeless), media and advertising exposure, and KAP.

The survey was developed in English, translated into Hindi, and tested for readability and accuracy by both a local clinician and layperson. The survey was then piloted over a two-day period and adapted accordingly. To maximize the consistency between researchers a one-day orientation was given to them.

The primary outcomes of interest of this study were as follows:

- prevalence of tobacco consumption: current use of tobacco was defined as any use in the last month, an ex-user as having used tobacco in the past, but not in the last month, and never smoker as having never used tobacco in any form;
- awareness levels of the dangers of tobacco;
- attitudes towards tobacco usage and quitting;
- practices around tobacco use and quitting.

The research team collected the data on paper forms (questionnaire) and then entered deidentified data into STATA. Only one out of 1854 forms was inadequately completed and this was removed from the sample. The data was then cleaned and inconsistencies checked against the original forms. Various subanalyses on tobacco usage were tabulated and prevalence was analyzed by* income level, occupation, age, and sex and numbers cohabitating*. Estimates were accompanied by 95% confidence intervals calculated using standard formulas and incorporating assumptions in relation to the clustering effect by village and residence unit. These* clustering effects* were taken into account in all the appropriate analyses.

### 2.3. Ethical Issues

Ethics approval was also obtained from the Alfred Health Human Ethics Committee, Alfred Health, Australia, and the Chamba Hospital Ethics Committee (India). An Information and Consent Form was developed, translated into Hindi, and approved by both ethics committees. Additionally, upon entering a village, the team would sit with the village head (Pradhan), other village members, and the community health workers in order to answer any questions. There is little, or no, perceived risk to the participants. Data did not contain identifiable information.

## 3. Results

(*More detailed data is available on request but there was inadequate space to present it all here.*)

### 3.1. Subjects/Participants

The study sample size was 1853 people, including 927 men and 926 women. These participants were spread amongst 15 clusters and selected from a total sampling frame of 195354 people.

The villagers were similar in many respects with high illiteracy, poor quality housing, and widespread poverty. The demographic data demonstrated a similarity between the clusters in terms of sex ratio, age profile, education status, and occupational profile. This suggests a low intercluster variance.

Among all current tobacco users the overall prevalence was 38.9%. However, 69.5% of males used tobacco and 54.0% smoked tobacco. 96.3% of women were not tobacco users compared with only 46% of males (see Table 1). A current user (for both smoked and nonsmoked forms) was considered to be a user if they had used any at all in the last month. An ex-smoker or ex-user of chewed forms was a person who had smoked/or chewed tobacco in the past, but not in the last month.

#### 3.1.1. Tobacco Usage Prevalence across Different Demographics

The prevalence of people smoking increases as age increases, with rates in the oldest age category (>68 years old) being around double that in the youngest category (18–34 years old). However, the majority of the burden of tobacco usage (64% of users) existed in the younger age categories (<52 years old). That is, although the rates were higher in the older ages there were less people alive in these age groups. Interestingly prevalence of chewing actually decreased with age perhaps suggesting substitution with smoked forms of tobacco (Table 2(b)).

Lower education status was also associated with an increased prevalence of tobacco usage, with 42.1% of those with no education using tobacco, 40% of those who reached upper primary, and only 29.9% of those who had completed higher studies. The association between smoking and prevalence was strengthened when controlling for other factors through the regression analysis.

As shown in Table 2(a), in relation to occupation, the mean prevalence of current tobacco users was among drivers (76.6%), labourers (75.1%), and government workers (62%). Students used tobacco at lower rates (22%) whilst occupations undertaken mainly by women had low prevalence (houseworkers (8.9%), agricultural labourers (26.2%)). Surprisingly there is no clear relationship between household income and tobacco usage.

A logistic regression analysis which incorporated sex, age group, occupation type, education level, and income group was conducted to predict tobacco use and was found statistically to be significant against a constant only model (chi square 947.2, *P* < .001 with df 20). The Wald criteria demonstrate that sex, age group, and education made a significant contribution in prediction of tobacco usage (*P* < 0.05). The odds ratio indicates that females are less likely to smoke or chew tobacco compared to males (OR 0.04, 95% CI 0.02–0.05); similarly a younger age group (i.e., 18–34 years) is less likely to use tobacco compared to the oldest age group of >68 years (OR 0.43, 95% CI 0.23–0.80) and people who have completed higher studies tend to use tobacco less compared to people with no education (OR 0.36, 95% CI 0.23–0.58).

*Attitudes and Practices towards Tobacco Use*. 97.9% of those surveyed wanted less tobacco usage in their villages, including 97.6% of users and 98.1% of nonusers. 70% of current users wished to quit and of those who did not want to quit 58% wanted to cut back. 82.4% of those surveyed, including 83.5% of users and 81.7% of nonusers, supported clear and prominent health messages on tobacco products.

87% of tobacco users were aware that tobacco was harmful to health and awareness was higher amongst males (89.7% versus 77% in females), the young (85% in those 18–34 year olds versus 51% in >68 year olds), and educated (93.9% if higher education versus 75.4% if primary educated only). See Table 3.

*Awareness That Smoking Causes Serious Harm across Different Education/Sex/Age*. Interestingly males, despite using tobacco at much higher rates, also tended to have more awareness about the dangers of tobacco (Table 3). As expected those with lower formal education levels tended to have lower awareness of all dangers. Those who were older (especially over 68) had much lower levels of awareness than younger groups.

Reasons for not stopping were that they use tobacco to relieve stress (64%), they simply like using (43.5%), cravings make it difficult to stop (43.5%), and they spend time with tobacco users (37.8%). 14% stated that peer pressure was a barrier to quitting.

**Figure 1:**
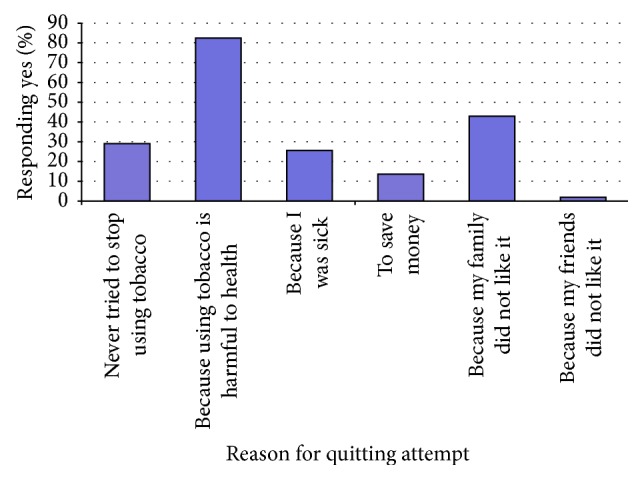
Factors that promoted attempts to stop using tobacco.

*Assistance Given for Cessation*.* 27.4% of all tobacco users had attempted to stop using tobacco* and the stand-out reasons for the attempt were health reasons. 84% stated this as a main reason (see Figure 1). Of those who tried to stop using tobacco 35% received no assistance at all, 54% received advice, support, or encouragement from family and friends, and 11% advice/support/encouragement from a health practitioner or chemist. Only 1% received medicines from a medical doctor and a further 1% from traditional doctors. None of the smokers had been given assistance in the form of nicotine replacement therapy.

*Advertising and Marketing Messages*. Survey participants were obtaining information about the dangers of tobacco through a variety of media especially from television (85%). In contrast, as shown in Table 4, very few people noticed tobacco promotions, with the exception of in TV/films (25.7%), on packaging (17%), and at stores (16%).

There were no significant differences between the males and females in regard to where they obtained information on the danger of tobacco and where they saw tobacco advertisements.

## 4. Discussion

The analysis of this survey indicates a large burden of tobacco usage in the state of Uttarakhand with 38.9% being current users of tobacco: 28% smoked and 21% using nonsmoked forms. We know that tobacco smoking kills one in three of long term users [15, 16]. Therefore, these very high tobacco usage rates represent a time bomb in Uttarakhand as tobacco associated morbidity and mortality is increasingly experienced over the coming decades. The rate in this study is higher than the national average (35%) and higher than that previously estimated in Uttarakhand (31%) [7]. However, this is consistent with other studies which consistently demonstrate higher rates of overall tobacco use in rural areas compared to urban areas [7, 14, 17].

In the general population smokeless tobacco products are the most commonly used (21% of the population) compared with smoked forms (9% of the population), whilst one-seventh (5% of the population) use both [7]. Our figures display a much more harmful pattern in Uttarakhand where more people use smoked forms than nonsmoked forms. Whilst chewing and smoking both have harmful effects, tobacco smoking has a far greater impact on mortality than do nonsmoked forms. Tobacco related cancers, heart disease, stroke, and other ill effects are far greater with smoked forms of tobacco than nonsmoked forms.

This combined prevalence figure masks an astounding prevalence amongst males whereby 69.5% of males use tobacco, 54.0% smoke, and 36.7% use nonsmoked forms. This survey shows that 93% of all smokers in Uttarakhand are male indicating that this is a male epidemic. These findings support an approach to tobacco control in Uttarakhand that predominantly targets young males. This discrepancy is consistent with GATS; however the gross prevalence amongst males in Uttarakhand (69%) is far higher than the national average for males (48–57%) [7]. Prevalence of tobacco smoking amongst men in Uttarakhand (54.0%) is more than double the national average of 23%, as recently estimated in a national survey undertaken in the same year [8]. The gender difference is particularly pronounced amongst tobacco smokers (54% in men versus 3.7% in women) due to cultural norms in India which discourage women from smoking [18]. In countries of South Asia, particularly India, traditional values tend to discourage smoking by the young or by women, but there is no such taboo against using smokeless tobacco. Thus, most women who use tobacco use it in smokeless forms. Tobacco use, in whatever form, generally begins during adolescence.

Consistent with other surveys such as the GATS, the analysis describes higher tobacco usage rates in older cohorts [7, 19]. This could reflect that younger people are now using less tobacco; however given the extremely low rates of ex-users it more likely reflects accumulation of users over time. Additionally, we found higher rates amongst the labourers and the least educated in the population, a finding that is consistent with most countries [7, 14, 20–23]. In Uttarakhand, after controlling for age/sex/income/education level there was a three times higher rate of tobacco usage amongst the least educated when compared with the most educated. This is consistent with India-wide studies that show those with no education are three times more likely to smoke, and almost twice as likely to use smokeless tobacco, compared to those with a postgraduate education [14, 20, 23]. This reinforces the need to focus tobacco control messages at lower education levels in Uttarakhand.

### 4.1. Knowledge of Harms

Despite the high levels of illiteracy, rurality, and poverty in Uttarakhand, nearly all respondents were aware that smoking was injurious to health (89.3%) and harmed others (73.5%). In contrast, few were aware that smoking causes infertility (32% awareness) and strokes (12% awareness). This lack of awareness is important because awareness of infertility and birth defects might be more impactful for younger people than ill-health in the distant future would. The mediums for most tobacco control campaigns have poor penetration into poor and uneducated demographics [7]. In this study those who were female, uneducated, and older had lower awareness that tobacco was harmful (see Table 3). There is solid evidence in that graphic rotating warnings on packages would be an effective intervention to raise awareness about the myriad of diseases caused by tobacco [24, 25]. Interestingly, 85% of our study group, and more than 83.5% of tobacco users, wanted better health warnings on tobacco packages. This positive attitude towards better health warnings is consistent with other national studies such as Arora et al., 2013 [24].

### 4.2. Advertising and Marketing

Tobacco promotion and advertising increase tobacco usage as indicated by Global Youth Tobacco Survey data demonstrating that youth exposed to cigarette advertising through sports events, televised events, newspapers/magazines, and free cigarettes promotions were significantly more likely to be smokers [26]. Conversely, youth exposed to antismoking media messages were less likely to be current smokers [26]. The same media forms are used to both promote tobacco and protect people from it.

This study indicates that in Uttarakhand we may be winning the advertising and marketing war as few noticed tobacco advertising information whereas most noticed warnings in the media. In Uttarakhand, advertising is banned except at point of sale and packaging. With the exception of shops where tobacco is sold there is little exposure to adverts which partly explains why advertising was often unnoticed. In regard to television and film, which are particularly effective at promoting behaviours, only 25.7% noticed advertising of tobacco products (presumably they are referring to their stars smoking in films) whereas nearly everyone noticed warnings about tobacco. Tobacco advertising bans and limits on showing tobacco usage in Bollywood movies are having an impact (COTPA, 2003). Decreasing exposure to advertising is very important to limit recruitment of young tobacco users. A recent study of 4,000 adolescents in New Delhi showed a significant association between exposure to tobacco in Bollywood movies and students' own tobacco use [27]. Further, analysis of the third National Family Health Survey indicates attending the cinema once a month or more increases the likelihood of an individual either smoking (both males and females) or chewing (males only) tobacco [28].

### 4.3. Recommendations for Tobacco Control Interventions

What is clear from this study is that additional tobacco control interventions are necessary and desired by the community with both users and nonusers wanting less tobacco use (97.8%) and 82.4% favouring more graphic health warnings. At an individual level the majority of users (70%) wanted to stop and 58% of those who did not want to stop at least wanted to cut back. This appetite for controlling tobacco in Uttarakhand potentially provides an enabling sociopolitical environment and should support the implementation of government tobacco control initiatives currently being considered. However, currently little help is available to aid quitting with only 1 in 10 obtaining professional input and 1 in 100 receiving medications to help them quit. Such help has been shown to increase quitting successes. Cessation programs and interventions that provide assistance are urgently required if we are to mitigate the Uttarakhand tobacco usage epidemic.

Doctors need to be trained in providing cessation advice and increasing awareness as in this study only 1% of those trying to quit received any assistance from a doctor. Where doctors are difficult to access, as is the case in these mountain areas, health workers such as accredited social health activist (ASHA) workers need to be similarly trained. Community health workers, from both government and NGOs, are well placed to be tobacco control advocates in their local community.

Various results suggest that a community based information and awareness campaign might be a timely intervention in Uttarakhand. Interestingly, ex-smokers (2%) and ex-tobacco chewers (0.5%) are few which indicates that having initiated, very few stop using tobacco. When considered alongside the finding that only a quarter have ever attempted to quit this may represent* low hanging fruit* in Uttarakhand. That is, it is reasonable to expect a good number to quit when simply informed fully of the tobacco impact. Studies have shown that where prevalence is high and number of ex-smokers is low, then with simple awareness campaigns a large number of smokers will simply choose to quit. Any awareness campaigns should focus on the health impacts on users, their children, and their community because for those who had attempted to quit their health problems were the most important motivating factor.

Tobacco control efforts should target youth, given that peer pressure was important in promoting initiation of tobacco (this study) and 40% of all tobacco users in India initiate before 18 years of age (GATS) [7] and the mean age of initiation of tobacco use amongst users aged 20–34 years was 17.9 years old. Therefore, behaviour change counselling activities, counselling, and quit-line programmes need to be started at schools and colleges and at community level for drop outs and illiterates. After initiation of tobacco use, overcoming addictive behaviours is more difficult (43.5% named cravings as a barrier to quitting).

### 4.4. Limitations

Smoking and chewing status was by self-report, and taboo about tobacco use, particularly in females, might have resulted in underreporting. Additionally, smoking rates were highest amongst drivers, labourers, and government workers and as these professions are based away from the home they may have been underrepresented due to the methodology of selecting from those present at home. However, this bias would lead to an underestimating the size of the problem. Generalisability beyond Uttarakhand is difficult given that the sampling frame was limited to India. Only two districts were included due to time and financial constraints, but these were felt to reflect the demographics of other districts in Uttarakhand.

## 5. Conclusions 

This study demonstrates high prevalence of tobacco usage, particularly smoked forms, amongst men in the mountains of North India. Very few users have successfully quit and yet both tobacco users and nonusers supported tobacco control in their community. These findings substantiate the need for development and implementation of a tobacco control program in the area. A proposal to initiate such a community based program is being discussed with the Uttarakhand government and other partners. These study results will permit high quality, integrated, and cost effective tobacco control programs in North India.

## Figures and Tables

**Table 1:** Prevalence of tobacco usage in Uttarakhand.

	Female (%)	95% CI	Male (%)	95% CI	Overall (%)	95% CI
Current tobacco users (both smokers and chewers)	**9.4**	(6.4, 12.4)	**69.5**	(63.2, 75.8)	**38.9**	(35.3, 42.7)
Current tobacco smokers	**3.7**	(1.2, 6.1)	**54.0**	(42.1, 66.0)	**28.5**	(23.6, 33.4)
Current tobacco chewers	**5.9**	(2.0, 9.7)	**36.7**	(29.9, 43.6)	**21.0**	(16.3, 25.8)
Ex-smokers	**0.5**	(0, 1.09)	**3.2**	(0.0, 6.7)	**1.8**	(0.0, 3.8)
Ex-tobacco chewers	**0.1**	(0, 0.29)	**1.1**	(0.0, 2.5)	**0.6**	(0.0, 1.3)

**(a):** 

Occupation	(%)	95% CI	Education level/students	(%)	95% CI	Income level^*^ (Rs.)	(%)	95% CI
Agriculture	26.2%	21.3–31.1	Higher studies	30.0%	22.3–37.4	**0**–**2000**	32.7%	27.7–37.6
Driver	62.2%	42.4–81.9	Higher Secondary	40.6%	31.7–49.5	**2001**–**4000**	39.2%	36.1–42.3
Government	76.6%	54.0–99.2	Upper primary	40.2%	35.1–45.2	**4001**–**6000**	39.9%	34.9–44.9
Labourer	75.1%	69.0–81.2	Primary	40.0%	33.3–46.7	**6001**–**8000**	44.3%	33.7–54.9
Housework	8.96%	5.2–12.8	None	42.2%	35.5–48.8	**8000+**	39.3%	28.6–49.9
Shopkeeper	57.2%	46.2–68.1						
Student	22.6%	11.2–34.1						

^*^Household income is commonly estimated by food expenditure in Rupees/month.

**(b):** 

Age	Prevalence of tobacco usage (smoking or chewing)	95% CI	Prevalence of smoking	95% CI	Prevalence of chewing	95% CI	Prevalence of both chewing and smoking	95% CI
18–34	25.7%	22.6–28.8	16.5%	13.9–19.16	16.9%	14.3–19.6	7.7%	5.9–9.6
35–51	41.0%	37.8–45.7	31.6%	28.0–35.4	17.2%	14.2–20.3	7.1%	5.1–9.2
52–67	54.2%	49.0–59.4	47.4%	42.2–52.2	10.7%	7.5–13.9	3.8%	1.9–5.8
>68	55.9%	46.5–65.2	45.9%	35.5–55.3	14.4%	7.8–21.0	4.5%	0.6–8.4

**Table 3:** Awareness about risks/harms of tobacco usage in subgroups (percentage of those who were aware of the risks).

	Passive smoking (%)	Stroke association (%)	Infertility (%)	Heart disease (%)	Lung cancer (%)
Overall % who were aware of harms	73.5	40.5	32.5	67	89.3
Age group					
18–34	75.9	44.6	42.5	74.1	97.5
35–51	78.5	36.7	28.5	63.7	92.1
52–67	65.0	26.2	17.4	62.9	91.1
68>	46.3	15.5	13.4	29.5	88.8
Gender					
Male	80.0	41.7	33.4	72.1	93.0
Female	67.0	33.7	33.7	62.0	85.5
Education status					
Higher studies	85.1	58.8	46.1	90.2	98.0
Higher secondary	87.2	49.9	41.7	83.0	97.3
Upper primary	75.8	43.7	40.2	76.2	97.2
Primary	68.2	41.7	29.7	56.6	92.2
No formal education	63.1	18.6	20.1	49.0	77.4

**Table 4:** Source of advertising of tobacco products and information about the dangers.

Medium	Warnings/demotions			Tobacco promotions		
	Yes (%)	No (%)	Unsure	Yes (%)	No (%)	Unsure
Newspaper/magazines	46.5	37.5	16	4.3	74.8	12.9
Television/films	**85.9**	5	9.1	25.7	52.9	21.5
Radio	22.2	57.5	12.3	0.6	74.2	25.2
Billboards/posters/signage	33.8	44.6	21.6	0.3	75.2	24.5
Public transport vehicles	41.7	36.6	21.7	0.2	75.6	24.2
On tobacco packaging	18	33.8	48.2	12.9	68.4	18.7
Talking to family	41.7	40.5	17.8	N/A	N/A	N/A
Talking to friends	15.5	45.8	38.7	N/A	N/A	N/A
Stores where products are sold	N/A	N/A	N/A	16.2	65	18.8

## Appendix

Code Number
  —/—/— (Village (00-30)/Family (00-99)/Participant (00-99))
Age
  — (Estimated Years)
Date
  —/— (Day/Month)
Occupation
  —
Caste (or Religion)
  —
Sex: Female/Male
Highest Educational Attainment:
  None/Primary/Junior High/High school/Intermediate/College (Up to 5th/8th/10th/12th)
How many people live in your house? (currently)
  —

*All Participants*

(1a) Based on what you know or believe, does smoking tobacco (such as cigarettes, bidis, hukkah, cigars or pipes) cause serious illness?
  (Yes/No/Don't Know/Refused)
(1b) (If yes) Which of the following illnesses?
  - Lung and throat cancer
    - (Y/N/DK/R)
  - Heart disease
    - (Y/N/DK/R)
  - Infertility
    - (Y/N/DK/R)
  - Stroke
    - (Y/N/DK/R)
  - Harm to non-smokers who are in the same room and breathe in the smoke
    - (Y/N/DK/R)
  - Other…please specify
    - —
(1c) Based on what you know or believe, does using smokeless tobacco (such as paan, khaini or gutkha) cause serious illness?
  (Y/N/DK/R)
(2) How many rupees does your household spend on food each month? (circle)
  0–1000
  1001–2000
  2001–3000
  3001–4000
  4001–5000
  5001–6000
  6001–7000
  7001–8000
  8001–9000
  >9001
(3) Have you ever smoked tobacco? (circle)
  Current/ex-smoker/never
  (*current— smoked any amount in the last month, ex-smoker—If the patient smoked any in the past, but not in the last month*)
If yes then
  — (Per day/week/occasional)
(4) Have you ever used smokeless tobacco? (circle)
  Current/ex-user/never
  (*current—used tobacco (non smoked forms) in last month, ex-user—used tobacco (non-smoked) in past, but not in last month*)
If yes then
  — (Per day/week/occasional)

*If the Participant Currently Uses Tobacco of Any Form*…

(5) How many rupees have you spent on tobacco for yourself in the past month? (circle)
  0
  1–150
  151–300
  301–450
  451–600
  601–750
  751–900
  901–1050
  >1051
(6) Would you like to give up using tobacco?
  (Y/N/R)
(If yes) How soon? In the next
  month/year/not sure
(If no) Would you like to reduce how much tobacco you use?
  (Y/N/R)

*If the Participant Has Ever Used Tobacco*…

(7) How many years ago did you start using tobacco? (*Number of years ago*)
  —
(8) - Have you ever tried to stop using tobacco?
    - (Y/N/R)
(If yes) When you attempted to stop, did you try any of the following to help you?
  (b) Receiving advice, support or encouragement from family and friends
    (Y/N/DK/R)
  (c) Receiving advice, support, encouragement from a health practitioner or chemist
    (Y/N/DK/R)
  (d) Nicotine replacement therapy, for example, patches, gum or lozenges
    (Y/N/DK/R)
  (e) Medicines from a medical doctor
    (Y/N/DK/R)
  (f) Traditional medicines to help stop using tobacco
    (Y/N/DK/R)
  (g) Other…please specify
    —
(9) I will now give you a list of common reasons why people find it difficult to stop using tobacco. Do/did any of these apply to you?
  (a) Because most of the people I spend time with also use tobacco
    (Y/N/DK/R)
  (b) Because of pressure from friends, or wanting to fit in with friends
    (Y/N/DK/R)
  (c) Because I like using tobacco too much
    (Y/N/DK/R)
  (d) Because using tobacco helped me to relieve stress or negative moods
    (Y/N/DK/R)
  (e) Because of cravings or physical urges to use tobacco
    (Y/N/DK/R)
  (f) Because I do not believe that using tobacco is bad for health
    (Y/N/DK/R)
  (g) Other…please specify
    —
(10) - Have you ever attempted to stop using tobacco?
    - (Y/N/R)
(If yes) What were the main reasons you decided to stop?
  (b) Because using tobacco is harmful to health
    (Y/N/DK/R)
  (c) Because I was sick
    (Y/N/DK/R)
  (d) To save money
    (Y/N/DK/R)
  (e) Because my family did not like it
    (Y/N/DK/R)
  (f) Because my friends did not like it
    (Y/N/DK/R)
  (g) Other…please specify
    —

*All Participants*

(11) I will now give you a list of options. Please tell me if any of these are places you have noticed information about the dangers of tobacco in the past 30 days?
  - Newspapers/magazines?
    - (Y/N/DK/R)
  - Television?
    - (Y/N/DK/R)
  - Radio?
    - (Y/N/DK/R)
  - Billboards/Posters/Signage?
    - (Y/N/DK/R)
  - Cinemas?
    - (Y/N/DK/R)
  - Public transport vehicles?
    - (Y/N/DK/R)
  - When talking to your family?
    - (Y/N/DK/R)
  - When talking to your friends?
    - (Y/N/DK/R)
  - On the packaging of tobacco products?
    - (Y/N/DK/R)
  - Somewhere else? (specify)
    - —
(12) Are any of the following options places where you have noticed advertising of tobacco products in the past 30 days?
  - Stores where products are sold?
    - (Y/N/DK/R)
  - On the packaging of tobacco products?
    - (Y/N/DK/R)
  - Newspapers/magazines?
    - (Y/N/DK/R)
  - Television?
    - (Y/N/DK/R)
  - Radio?
    - (Y/N/DK/R)
  - Billboards/Posters/Signage?
    - (Y/N/DK/R)
  - Cinemas?
    - (Y/N/DK/R)
  - Public transport vehicles?
    - (Y/N/DK/R)
  - Somewhere else? (specify)
    - —
(13) Would you like to see less tobacco use in your village or community?
  (Y/N/DK/R)
(14) Are you in favour of having clear and prominent messages on all tobacco products that warn users about the dangers of tobacco?
  (Y/N/DK/R)
(15) Field Notes (e.g., please comment on overcrowding, types of animals/pets, supply of tobacco).
